# rTMS Therapy Reduces Hypofrontality in Patients With Depression as Measured by fNIRS

**DOI:** 10.3389/fpsyt.2022.814611

**Published:** 2022-06-22

**Authors:** Yasuo Kawabata, Shin-ichi Imazu, Koichi Matsumoto, Katsunori Toyoda, Makoto Kawano, Yoichiro Kubo, Shinya Kinoshita, Yoshitaka Nishizawa, Tetsufumi Kanazawa

**Affiliations:** ^1^Department of Neuropsychiatry, Osaka Medical and Pharmaceutical University, Osaka, Japan; ^2^Stanford University, Stanford, CA, United States; ^3^The Florey Institute of Neuroscience and Mental Health, University of Melbourne, Melbourne, VIC, Australia

**Keywords:** cerebrovascular circulation, depression, functional near-infrared spectroscopy, oxyhemoglobins, prefrontal cortex, repetitive transcranial magnetic stimulation

## Abstract

Multichannel functional near-infrared spectroscopy (fNIRS) is a tool used to capture changes in cerebral blood flow. A consistent result for depression is a decrease in blood flow in the frontal cortex leading to hypofrontality, which indicates multidomain functional impairment. Repetitive transcranial magnetic stimulation (rTMS) and elective convulsive therapy (ECT) are alternatives to antidepressant drugs for the treatment of depression but the underlying mechanism is yet to be elucidated. The aim of the current study was to evaluate cerebral blood flow using fNIRS following rTMS treatment in patients with depression. The cerebral blood flow of 15 patients with moderate depression after rTMS treatment was measured using fNIRS. While there was clear hypofrontality during pre-treatment (5 ± 2.5), a notable increase in oxygenated hemoglobin was observed after 30 sessions with rTMS (50 ± 15). This increased blood flow was observed in a wide range of channels in the frontal cortex; however, the centroid values were similar between the treatments. Increased blood flow leads to the activation of neuronal synapses, as noted with other neuromodulation treatments such as electroconvulsive therapy. This study describes the rTMS-induced modulation of blood oxygenation response over the prefrontal cortex in patients with depression, as captured by fNIRS. Future longitudinal studies are needed to assess cerebral blood flow dynamics during rTMS treatment for depression.

## Introduction

Brain activity assessed by functional near-infrared spectroscopy (fNIRS) *via* blood oxygen change has been utilized to distinguish psychiatric disorders, such as depression, bipolar disorder, and schizophrenia (1, 2). In particular, a number of reports have repeatedly shown reduced cerebral blood flow during the verbal fluency task (VFT) in patients with psychiatric disorders compared to healthy controls (3–6). Since the classification of psychiatric disorders is currently based on expressed symptoms, clinicians urgently need relevant biological markers to add persuasiveness to their practice. Multichannel fNIRS is a non-invasive neuroimaging technique for observing the living brain during tasks; therefore, it has a strong potential to distinguish major psychiatric disorders. Repetitive transcranial magnetic stimulation (rTMS) is efficacious for treating depression (7). A meta-analysis of the efficacy of rTMS reported its robustness in reducing the severity of depression (8). Moreover, this technique is easier to adopt in practice compared to electroconvulsive therapy (ECT), which requires general anesthesia and an operating room (9). These two treatment techniques are similar in terms of the direct electrostimulation of the brain (10). While the underlying mechanism of action for neuromodulation therapies (ECT and rTMS) is yet to be determined, the role of medication therapies in increasing monoamine levels at the synapse has been suggested (11, 12). Since neuromodulation and medication therapies have different durations of response, different modes of action are postulated.

A recent finding suggested that fNIRS is a trait marker, which refers to alterations in functioning that persist in those who have experienced major depression when they are no longer depressed, rather than a state marker, which is characteristic of the clinical status, because it reveals consistent hypofrontality during antidepressant medication in drug-naive patients with major depressive disorder (MDD) (13). Measuring oxygenated hemoglobin (oxy-Hb) in the VFT showed the same activation between pre-and post-drug treatment despite the significantly improved severity of MDD (14). However, to date, only a few studies have used fNIRS to evaluate rTMS as a treatment for MDD, probably because rTMS has been developed primarily in North America, whereas fNIRS has been mainly studied in Japan and other South Asian countries. The main aim of the current study was to evaluate hypofrontality using fNIRS, for the comparison of the pre- and post-rTMS treatment of depression.

## Materials and Methods

### Participants

We noticed the enrollment to rTMS therapy in community patients, therefore the recruited participants were from other outpatient clinics. Fifteen patients with moderate MDD were included in this study (seven men and eight women). These participants had received more than two antidepressant medication treatments before rTMS; however, a satisfactory response was not achieved. MDD diagnosis was based on an interview with the Structured Clinical Interview for DSM (SCID) (15) by blinded clinical psychologists. Patients with intellectual disability, impaired language disability, or difficulty speaking Japanese were excluded in this study. The demographic data are presented in Table 1; the mean years of medication was 6.9 (SD ± 4.9). All patients were right-handed. The mean equivalent dose of imipramine (antidepressant), chlorpromazine (antipsychotic), and diazepam (benzodiazepine) was 192 ± 122, 67 ± 108, and 10.2 ± 8.6 mg, respectively. During the rTMS procedure, the medication was not changed. Ethical approval for this research was obtained from the committee of the Osaka Medical College (IRB-approval number: 970-1). All participants gave oral approval and written informed consent.

**Table 1:** Demograhic data.

Subjects	15
Age (years)	42.87 ± 8.93
Gender	Male 7 Female 8
WAIS-III (Full IQ)	96.40 ± 19.60
Age of onset	41.2 ± 8.86
Treatment period (month)	18.17 ± 14.15
MT level	1.12 ± 0.20
DSM-IV (SCID)	
Major depressive disorder, single episode	
296.21 Mild	1
296.22 Moderate	4
296.23 Severe without psychotic features	1
Major depressive disorder, recurrent	
296.32 Moderate	5
296.35 In partial remission	4
Comorbid disorders	
300.02 Generalized anxiety disorder	1
300.23 Social anxiety	2
300.82 Somatoform disorder NOS	1
Medication Equivalent dose (mg/day)	
Antidepressants (Imipramine-E.D.)	192 ± 122
Antipsychotics (Chlorpromazine-E.D.)	67 ± 108
Benzodiazepines (Diazepam-E.D.)	10.2 ± 8.6
E.D. = equivalent dose	
Dominant hand	Right 15 Left 0

### rTMS

**Figure 1:**
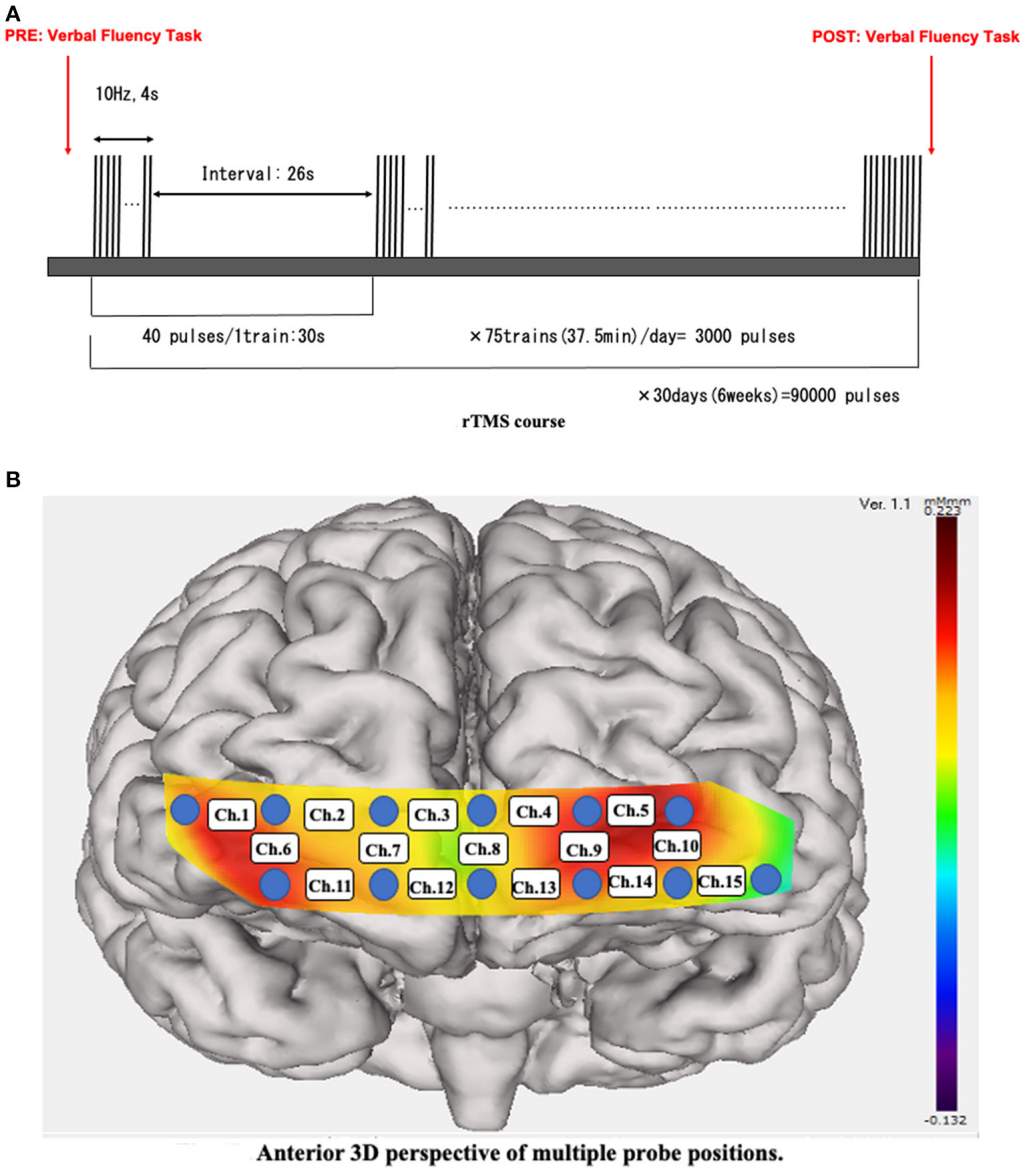
**(A)** rTMS course. A 10-Hz pulse sequence was applied for 4 s followed by a 26-s interval. We applied 3,000 pulses per train for 75 trains per day over the course of hospitalization. rTMS, repetitive transcranial magnetic stimulation. **(B)** Anterior 3D perspective of multiple probe positions. Twelve probes (lowest probes according to the Fp1–Fp2 line) were placed on the anterior brain. The 15 channels were divided into temporal (Ch. 1, 6, 10, and 15) and frontal (the remaining channels) positions.

The NeuroStar^®^ device (16) (Neuronetic Inc., Pennsylvania, USA) was used for 30 days of stimulation. In the current protocol design, 5 days of continuous stimulation per week were performed for 6 weeks. A 10-Hz pulse sequence for 4 s followed by a 26-s quiet period at a 120% motor threshold was the generated stimulation. A daily session lasted 37.5 min in total, including 40 pulses per train, with 75 trains in 1 day (3,000 pulses) (17, 18) (Figure 1A). Stimulation was set at the left dorsolateral prefrontal cortex (DLPFC), placed 5.5-cm anterior to the motor threshold location (19, 20). All rTMS treatment procedures were performed according to the guidelines provided by the working group to formulate a policy for the proper usage of rTMS in the Japanese Society of Psychiatry and Neurology (21).

### Clinical Outcome Assessment and the Medication During the rTMS Course

**Figure 2:**
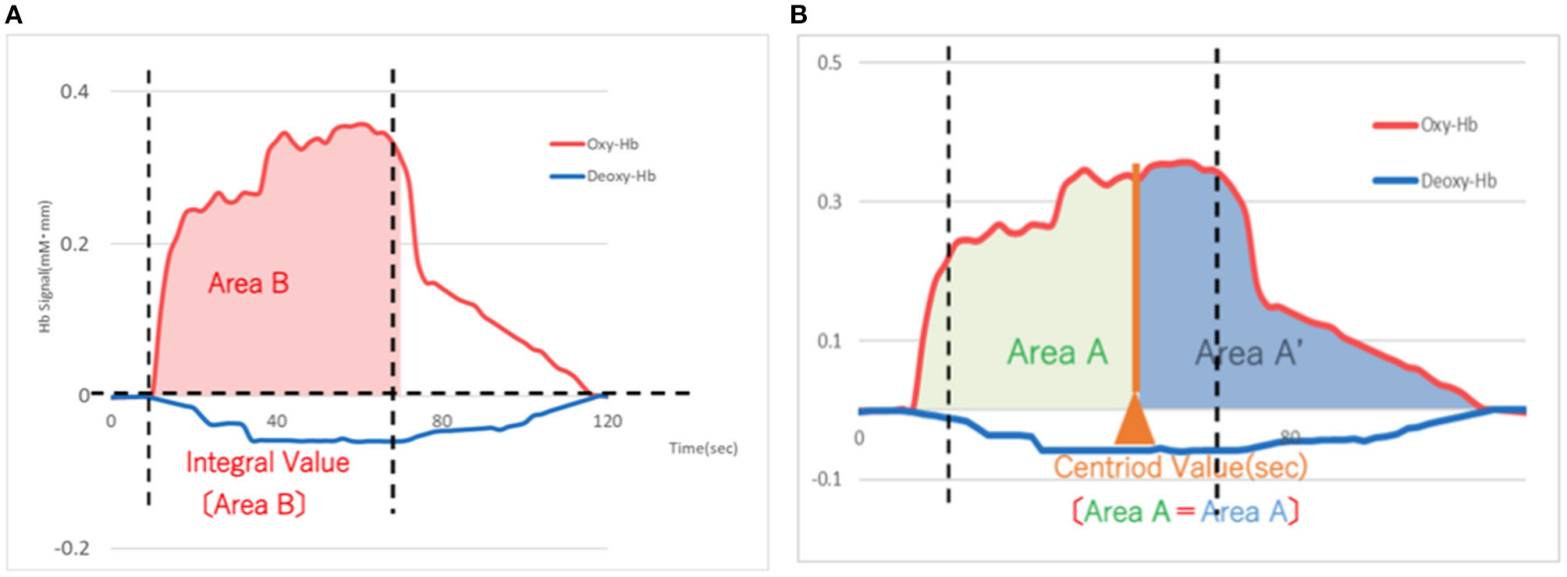
**(A)** Schema of the integral value of blood flow (Area B) based on the means of 15 patients with depression after rTMS treatment. The integral value of oxygenated hemoglobin (red) represents an increase in blood flow for each probe. X-axis: time (sec), Y-axis: Hemoglobin signal (mM-mm) rTMS, repetitive transcranial magnetic stimulation. **(B)** Schema of the centroid value (timing of activation) based on the means of 15 patients with depression after rTMS treatment. The centroid value of oxygenated hemoglobin (red) is the timing of activation on each probe. X-axis: time (sec), Y-axis: Hemoglobin signal (mM-mm) rTMS, repetitive transcranial magnetic stimulation.

**Figure 3:**
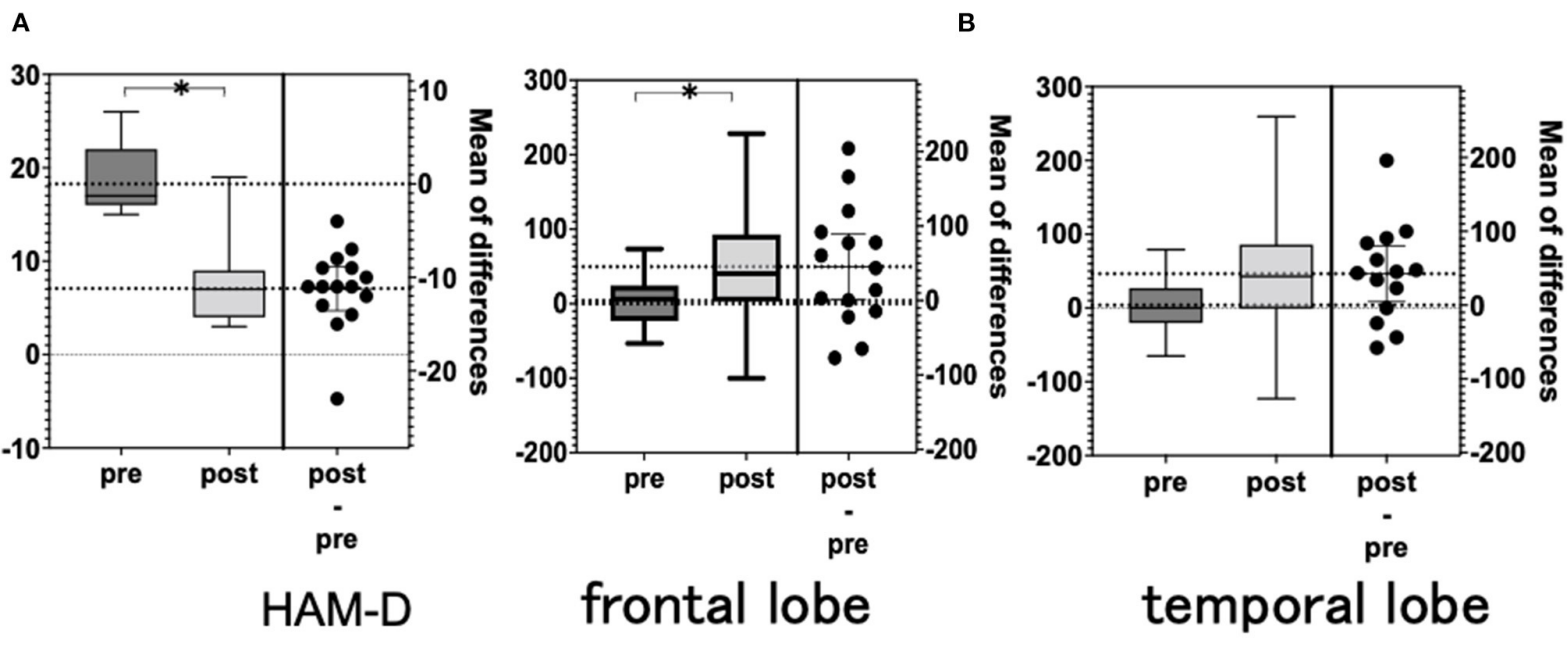
**(A)** Hamilton Depression Scale (HAM-D). **(B)** NIRS integral values (average). NIRS, near-infrared spectroscopy.

The Hamilton Depression Scale (HAM-D) (22) was used to assess the severity of depression. The assessment was performed by clinicians before starting the rTMS treatment (pre; blue in Figure 3) and after treatment (post; red in Figure 3). Antidepressant medication was not changed during the rTMS treatment, although sleeping pills were allowed as needed only a few times during the entire course. A 15-channel fNIRS (OEG-17ME; Spectratech Inc., Tokyo, Japan) with recording using a 12-probe device was used to assess changes in oxy-Hb and deoxy-Hb in the brain during the VFT. The full VFT procedure included a 30 s pre-task baseline, a 60 s VFT, and a 70 s post-task baseline. For the pre- and post-task baseline periods, participants were instructed to repeat aloud the five Japanese vowels (“a,” “i,” “u,” “e,” “o”). The subtraction method (task minus pre- and post-task baseline) minimized vocalization effects during the VFT. During the task, participants were instructed to generate as many Japanese words beginning with a designated syllable as possible. The three sets of initial syllables (1. /to/,/se/,/o/; 2. /a/,/ki/,/ha/; 3. /na/,/i/,/ta/) were presented in counterbalanced order among the subjects, and each syllable changed every 20 s during the 60 s task (23, 24). Briefly, the fixed fNIRS probes were 3 × 11 thermoplastic shells, and we set the lowest probes according to the Fp1–Fp2 line, along the international 10–20 system used in EEG (Figure 1B). Participants sat comfortably in a quiet, day-lit room. The task procedures were explained by a clinical laboratory technician who subsequently monitored head movement during the procedure. To avoid the effect of motion artifacts, data with clear evidence of head movement were omitted from further analysis. Similar to a widely used assessment method (2), the integral value of oxy-Hb and the centroid value of oxy-Hb were calculated for the frontal and temporal lobes using channel 2–14 and channel 1 and 15, respectively (Figures 2A,B). These were captured between pre- and post-VFT (Figure 1A). On the healthy controls measured by fNIRS, the oxyhemoglobin concentration is generally more pronounced in response to the VFT, increasing during VFT execution and reaching a peak value at the end of the VFT. Oxyhemoglobin then gradually returns to baseline values (25).The integral value of oxy-Hb is the value of frontal brain activation, whereas the frontal centroid value is the timing of activation and is often used to distinguish psychiatric disorders. A *t*-test was used to compare pre- and post-treatment values to detect the significance.

## Results

### HAM-D

The total HAM-D score was significantly lower after rTMS than before rTMS. The total HAM-D score decreased in 14 of the 15 patients but remained unchanged in 1 patient (Table 2 and Figure 3).

**Table 2:** The difference between pre- and post-rTMS treatment regarding the severity of depression (HAM-D) and fNIRS assessment.

	**Pre**		**Post**		***t*-stats**	***p*-value**		**df**
	**Mean**	**SD**	**Mean**	**SD**				
HAM-D	18.27	3.75	7.07	4.03	−10.14	<0.001	**	14
Integral value at frontal cortex	4.18	33.67	49.64	89.67	2.19	0.05	*	14
Centroid value at frontal cortex	57.61	21.18	51.63	41.28	−0.45	0.66	n.s.	14
Integral value at temporal cortex	4.01	41.32	46.44	90.75	2.42	0.03	*	13
Centroid value at temporal cortex	58.89	27.14	69.16	15.88	1.22	0.21	n.s.	13

^*^p < .05 ^**^p < .01

### fNIRS

**Figure 4:**
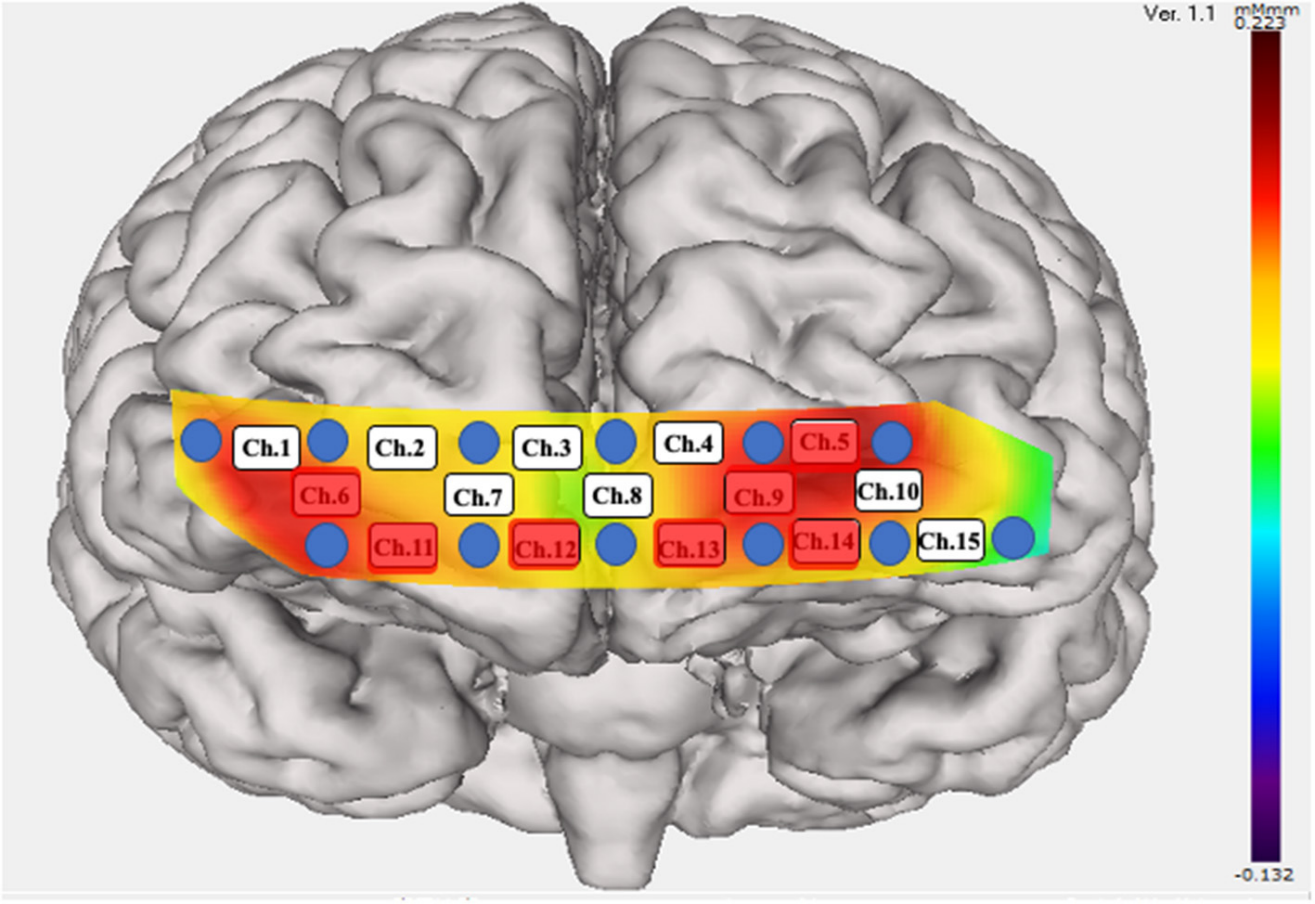
Significant increase in the integral value of blood flow. Twelve probes (lowest probes according to the Fp1–Fp2 line) were placed on the anterior brain. Fifteen channels were divided into temporal (Ch. 1, 6, 10, and 15) and frontal (the remaining channels) positions. A significant increase in blood flow was seen in the red colored probes.

There was a significant difference in the mean integral value at the frontal and temporal cortices when comparing pre- and post-rTMS (Figures 3A,B). However, there was no significant difference in the mean centroid values for both the frontal and temporal regions. The analysis of each channel showed a significant increase in the integrated values of frontal channel 5, 9, 11, 12, 13, and 14 and temporal channel 6 (Figure 4). A similar analysis of the centroid values showed significant increments at frontal channel 5, 11, 12, 13, and 14, and temporal channel 1 and 10.

## Discussion

Fourteen of 15 depressed patients showed improvement in symptoms after rTMS treatment. In addition, the integral value of NIRS measurements of cerebral blood flow before and after treatment showed an ~10-fold increase (5 ± 2.5 vs. 50 ± 15). Our previous research found that the severity of various psychiatric disorders was correlated with the integral value of the frontal lobe with fNIRS (*n* = 43) (23). The current data consisting of MDD showed symptomatic improvement; therefore, it is possible that this marked increase in the integral value in the frontal lobe was due to these effects.

### Earlier Studies

Initial studies utilizing both rTMS and fNIRS attempted to determine the physiological mechanisms underlying MDD. fNIRS observed simultaneously or immediately after rTMS demonstrated a significant change in the time course of oxy-Hb (26, 27). In those studies, researchers attempted to reveal the most beneficial conditions for brain stimulation. The combination of rTMS coil and fNIRS probes confirmed an intensity-dependent increase in oxy-Hb (27). However, in the current study, fNIRS assessment was compared between pre- and post-30 days of rTMS treatment at a 120% motor threshold intensity. This is because our primary aim was to observe dynamic oxy-Hb changes after successful rTMS treatment in patients with MDD. Fifteen patients with moderate MDD showed significant improvement in their depression symptoms without major adverse events. Several patients experienced scalp discomfort during early stimulation, although the discomfort disappeared within 10 stimulations.

Eschweiler et al. conducted a pilot study utilizing fNIRS and rTMS to reveal whether the putative therapeutic effect of rTMS depends on the hemodynamic dysfunction of the left DLPFC in patients with depression (*n* = 12) (28). To date, this is the only previous study that assessed rTMS with fNIRS at the DLPFC in the brains of depressed patients based on a recent systematic review (29). Their design differed significantly from that of the current study in terms of stimulation patterns, strength of stimulation, interval days, task during the procedures of fNIRS, the existence of sham controls, and instruments; therefore, it is impossible to precisely compare their findings with ours, although total Hb increased on the mirror drawing task at the position next to the coil position. This is partly similar to the current study, although future research is required to shed light on the complexity of fNIRS and rTMS in patients with depression. In contrast, a recent study demonstrated fNIRS recordings during stimulation (30). They analyzed 15 patients with depression. Their fNIRS device had only one channel; thus, it was difficult to detect detailed locations in the brain, but they suggested that an increase in hemoglobin at the end of treatment would improve the treatment effect.

### Hypofrontality and the Differences Between rTMS and Antidepressant Treatment

The main finding of the present study is the significant increase in the integral value of oxy-Hb during the rTMS consisting of 30 sessions. Of note, the integral value of oxy-Hb was increased during measurements of the VFT. Compared to a study utilizing antidepressant medication (13) (paroxetine, milnacipran, or mirtazapine), rTMS was different in terms of consistent hypofrontality despite similar improvements in depressive symptoms. Although they are similar MDD treatment options, direct electrical stimulation *via* ECT or rTMS (31) differ from monoaminergic (especially by serotonergic) reactions resulting from antidepressant medication. Our rTMS finding is in accordance with the ECT study (32) in terms of the increase in cerebral blood flow after treatment, unlike for the study utilizing antidepressant medication (13). More than 85% of cerebral glucose is used mainly by neuronal synaptic activity (33); therefore, the difference in blood flow between the two treatments indicates altered activity in the synapse. While antidepressant medication simply increases the monoamine (especially serotonin and noradrenalin) levels in the synapses, it has been suggested that neuronal connectivity *via* the reconstitution of synapses occurs in these two stimulation treatments (33). In particular, when cortical- limbic connectivity is measured, the functional connection increases rapidly (34). The left DLPFC was the region where rTMS was stimulated in this study, and we found a marked increase in blood flow as assessed by fNIRS. This suggests that much of the reconstitution of synapses occurs in the course of rTMS treatment, although we did not measure the connectivity assessed by fMRI in the current study. Moreover, it is known that the penetration depth by fNIRS stays close to the surface (typically in the range of 10–40 mm from the skull) (35). Meanwhile stimulation by rTMS reaches relatively deep into the brain. This is because the mechanism of action of rTMS is due to the generation of a magnetic field, which is known to have an effect as deep as the DLPFC (typically 20–40 mm from the surface of the brain) (36, 37). In the future, we aim to simultaneously observe blood flow and connectivity during rTMS treatment.

### Comparison Between rTMS and ECT

Generally, rTMS and ECT are the treatment options for treatment-resistant depression, which is defined as non-response to adequate doses of two different antidepressants taken for a sufficient duration of time. This may indicate that the two modulation treatments were differentially effective compared to the antidepressant medications. A meta-analysis of high-frequency left DLPFC rTMS for treatment-resistant depression yielded a weighted mean difference of 2.31, and an effect size of 0.33, compared to sham stimulations (38, 39). For patients with one or more unsuccessful antidepressant drug therapy, a recent guideline recommended rTMS as a first-line alternative treatment option rather than ECT based on more than 30 systematic reviews and meta-analyses (39). Another systematic review concluded that ECT was the most efficacious but least tolerated option, while rTMS was the best tolerated treatment for MDD. It is because of the side effect due to ECT, such as transient cognitive impairment, which could be induced by a massive increase in CBFV (cerebral blood flow velocity). The amount is increased by about a 100% (40, 41) while an increase of 3.6–5.6% of CBVF in the hemisphere is stimulated by rTMS (42).

## Limitations and Conclusion

This study had several limitations. First, our data were solely derived from two points within a short duration. Another potential limitation is the small sample size and the lack of a sham control design. In conclusion, this study described the rTMS-induced modulation of the blood oxygenation response over the DLPFC in patients with depression, as captured by fNIRS. Future longitudinal studies and the comparison between the sham controls are warranted to assess cerebral blood flow dynamics during rTMS treatment for depression.

## Data Availability Statement

The raw data supporting the conclusions of this article will be made available by the authors, without undue reservation.

## Ethics Statement

The studies involving human participants were reviewed and approved by Ethics Committee of Osaka Medical and Pharmaceutical University. The patients/participants provided their written informed consent to participate in this study.

## Author Contributions

YKa: study design, data acquisition, data interpretation, data analysis, and writing the first draft. S-iI and KM: study design, data acquisition, and data interpretation. KT: data interpretation and critics on the first draft. MK and SK: data acquisition, data interpretation, and critics on the first draft. YKu: data interpretation and data analysis. YN: data acquisition and data interpretation. TK: study design, data interpretation, data analysis, and writing the final manuscript. All authors have approved the final article should be true and included in the disclosure.

## Conflict of Interest

KT has received speaker's honoraria from Eisai. SK has received speaker's honoraria from Meiji Seika Pharma, Otsuka, Sumitomo Dainippon Pharma, EA Pharma, and Eisai. S-iI has received fees on advertising materials from Teijin. YN has received speaker's honoraria from Eisai. TK has received speaker's honoraria from Janssen, Meiji Seika Pharma, Otsuka, Shionogi, Sumitomo Dainippon Pharma, and Takeda Pharmaceutical Company, fees on advertising materials from Teijin, research grants from Ministry of Health, Labor and Welfare, and Health and Labor Sciences Research Grant (20GC1017 Head: Takefumi Ueno). The remaining authors declare that the research was conducted in the absence of any commercial or financial relationships that could be construed as a potential conflict of interest.

## Publisher's Note

All claims expressed in this article are solely those of the authors and do not necessarily represent those of their affiliated organizations, or those of the publisher, the editors and the reviewers. Any product that may be evaluated in this article, or claim that may be made by its manufacturer, is not guaranteed or endorsed by the publisher.
